# A digital twin–driven deep learning framework for online quality inspection in tobacco transplanting

**DOI:** 10.3389/fpls.2026.1716046

**Published:** 2026-02-04

**Authors:** Qiuyang Zhao, Erdeng Ma, Jian Zhao, Zekun You, Jiahui Liu, Dong Zhao

**Affiliations:** 1 School of Technology, Beijing Forestry University, Beijing, China; 2 Yunnan Academy of Tobacco Agricultural Sciences, Kunming, China; 3 Key Lab of State Forestry Administration on Forestry Equipment and Automation, Beijing Forestry University, Beijing, China; 4 State Key Laboratory of Efficient Production of Forest Resources, Beijing, China

**Keywords:** crop management, deep learning, digital-twin, online quality inspection, tobacco, transplanting

## Abstract

Tobacco transplanting quality inspection is crucial for tobacco production, as it directly affects crop yield and quality of tobacco leaves. Accurate transplanting status detection and assessment provide essential support for replanting decisions and transplanting machine optimization. Traditional methods rely on manual inspection, which suffer from high cost, low efficiency, and unstable results. To tackle the aforementioned issues, this paper proposes a Deep Learning and Digital Twin driven Online Quality Inspection Method for Tobacco Transplanting, which consists of four core modules: Transplanting Status Detection, Multi-sensor Data Fusion, Digital Twin Visualization, and Operational Optimization Feedback. This paper proposes a lightweight improved YAN-YOLO11 algorithm capable of assessing normal, exposed-root, and buried seedlings. By fusing GNSS positioning data with visual detection results, the system estimates in-row spacing and performs status assessment for missed planting and double planting. The system establishes a virtual-real interactive closed-loop of “collection-detection-mapping-feedback” via the digital twin. By visualizing operational status in real-time and generating replanting path suggestions, it provides guidance for operation management and significantly improves inspection efficiency. Field experiments demonstrate that, compared with YOLO11n, YAN-YOLO11 improves precision and recall by 2.4% and 2.5%, respectively; mAP@50 increased by 3% to 80.9% ± 1.4%, and mAP@0.5:0.95 increased by 5.8% to 54.2% ± 1.0%, while significantly reducing model complexity. The system achieves a real-time performance of 30 FPS in the field, with an overall recognition accuracy of 90.74%, meeting practical application requirements. This study effectively enhances the digitalization, automation, and refined management of tobacco transplanting operations, providing a theoretical foundation and practical solution for the intelligent transformation of transplanting machinery and precision crop management.

## 1 Introduction

Tobacco, as one of the most important cash crops worldwide, has long occupied a significant position in the agricultural economies of many countries (Liu and Filippidis, 2024). In recent years, with the continuous expansion of the global market, the demand for both tobacco quality and yield has been increasing, and tobacco cultivation has been accelerating its transformation from traditional labor-intensive modes toward efficient mechanization, automation, and intelligence. Within the entire process of tobacco agricultural production, the transplanting stage is particularly critical, as its operational quality directly affects plant growth, final yield, and the quality of tobacco leaves (Basir et al., 2021; Sun et al., 2024). Accurate transplanting quality inspection allows for the timely detection of issues such as missed planting, double planting, and uneven in-row spacing, thus providing data support for replanting operations to improve land use efficiency. Furthermore, it identifies abnormal statuses like seedling burial and root exposure, offering essential evidence for evaluating the performance of transplanting equipment. However, current traditional methods for assessing the quality of tobacco seedling transplanting mainly rely on manual inspection and row-by-row counting. This approach is not only labor-intensive and time-consuming but is also constrained by the operator’s experience and attentiveness, making it prone to human error and difficult to ensure the accuracy and consistency of statistical results (Fan et al., 2018). Therefore, it is urgent to develop an efficient, objective, and automated transplanting quality inspection method to promote the deeper application of smart agriculture in the tobacco sector.

With the advancement of smart agriculture, digital technologies represented by digital twin and deep learning have been extensively studied and applied in agricultural production. The concept of the digital twin was first proposed by Michael Grieves in the early 21st century, initially applied in manufacturing and engineering, where physical systems were replicated through digital technologies for monitoring, prediction, and optimization (Sun et al., 2024). In the agricultural domain, its potential to improve precision farming and sustainability has been widely recognized (Escriba-Gelonch et al., 2024). Chen et al. (2023) proposed a digital twin and data-driven online monitoring method for transplanting machines in plant factories, which can assess transplanting effects in real time. By enabling real-time interaction between virtual mapping and physical equipment, this approach optimizes the transplanting process and effectively prevents quality issues caused by mechanical resonance. Li et al. (2025) developed a digital twin system combined with deep learning for monitoring beak deformities in caged laying hens. Through image recognition, the system provided real-time feedback on flock health status, thereby optimizing the early detection of beak deformities and improving management in poultry farming.

In crop cultivation, digital twins create virtual farmland models to simulate various crop management processes. By leveraging advanced technologies such as artificial intelligence and the Internet of Things (IoT), they can significantly enhance productivity and management efficiency in agricultural production (Nasirahmadi and Hensel, 2022). Wang et al. (2024) proposed an intelligent sugarcane breeding system based on artificial intelligence, blockchain, and digital twin technology, which enabled real-time data circulation and optimization, greatly improving breeding efficiency and shortening the breeding cycle. Zhang et al. (2023) designed a digital twin system for plant factories, which enabled real-time monitoring and regulation of multiple environmental variables within plant factories. This system optimized the control of plant growth environments and provided intelligent decision support for plant factory managers. Xu et al. (2025) developed a digital twin system for the growth process of winter wheat. By integrating UAV-based remote sensing and IoT devices, the system monitored the growth status of winter wheat in real time and provided accurate growth prediction and optimized management strategies.

In transplanting inspection, especially in research on seedling detection, deep learning and object detection have been widely applied due to their advantages of high accuracy and real-time performance. Cui et al. (2023) proposed a real-time missed-seedling detection and counting method for paddy fields based on YOLOv5s and the ByteTrack algorithm. Through a lightweight design, this method improved detection accuracy for small and overlapping seedlings and significantly reduced counting time. Vong et al. (2022) combined UAV imagery with deep learning models to propose a method for detecting maize emergence uniformity based on plant density, the standard deviation of plant spacing, and the average number of imaging days after emergence. They also developed a field mapping function, providing valuable references for farm-level decision-making. Liu et al. (2023) developed a maize emergence evaluation system based on UAV imagery and deep learning, in which a YOLO model was used to achieve efficient detection of maize seedlings and assessment of emergence uniformity. Furthermore, recent studies have also made progress in addressing detection challenges related to small objects and complex backgrounds. For example, the Seedling-YOLO model significantly enhanced the detection accuracy and speed for exposed seedlings and missing holes in broccoli transplanting by introducing ELAN-P modules and attention mechanisms (Zhang et al., 2024); meanwhile, the LSOD-YOLO algorithm optimized YOLOv8 through a cross-layer output reconstruction module, effectively resolving the missed detection of small objects while ensuring a lightweight design (Wang et al., 2025).

For tobacco transplanting seedling recognition, Shahid et al. (2024) proposed an aerial imagery-based tobacco plant counting framework. By integrating YOLOv7 with the SORT algorithm and overlapping detection, this framework achieved real-time and precise seedling detection and counting, significantly improving the efficiency and accuracy of crop emergence monitoring. In existing studies, most of them rely on UAVs or high-resolution remote sensing images for recognition. Although these methods offer higher acquisition efficiency, they lack sufficient coordination and real-time capability with transplanting operations. Zhang et al. (2022) designed a machine vision-based monitoring system for lodging and air-pocket conditions in rapeseed mat-type seedling transplanters. Mounted at the rear of the transplanter, the system collected and processed images in real time, effectively monitoring and assessing the operational status of the transplanter and improving the accuracy and timeliness of transplanting quality monitoring. Despite the achievements of the aforementioned studies, current tobacco transplanters primarily operate in an “open-loop” mode and generally lack integrated real-time perception capabilities regarding seedling posture, plant spacing, and transplanting status. Operators often cannot obtain immediate feedback on operation quality. Advanced image-based agricultural inspection systems have developed rapidly. For instance, Johansen et al. (2020) used UAV imagery to predict tomato biomass, and Jiang et al. (2022) utilized multispectral inversion for quinoa phenotyping. However, these methods mostly serve as “passive observers,” focusing on post-transplant offline monitoring rather than real-time interaction.

At present, the application of digital twin technology to tobacco transplanting quality inspection is still in the exploratory stage. For the specific requirements of tobacco transplanting, no comprehensive solution has yet emerged that integrates digital twins, visual recognition, data fusion, and real-time processing, making innovation and optimization at both the system architecture and algorithmic levels urgently necessary. How to realize transplanting quality inspection through automated approaches, reduce labor costs, improve inspection accuracy, and provide real-time feedback for equipment performance optimization and replanting operations has become an important research topic. To address these challenges, this paper proposes and develops a real-time tobacco transplanting quality inspection system that integrates deep learning and digital twin technology. The system takes the optimized YAN-YOLO11 object detection algorithm as its core, enabling high-precision detection of transplanted tobacco seedlings and accurate assessment of transplanting status. By incorporating multi-sensor fusion for precise in-row spacing estimation and combining it with an interactive digital twin visualization platform built on Unity3D, the system achieves real-time mapping between the virtual and physical fields, providing efficient decision support for precise replanting and operational optimization.

The main contributions of this paper are summarized as follows: 1. Multi-sensor Data Fusion: We achieved high-precision measurement of plant spacing and logical assessment of missed and duplicate planting by fusing GNSS positioning data with visual detection results. 2. Digital Twin Visualization and Feedback: A virtual-real interactive closed-loop was constructed based on Unity3D, which not only achieved real-time mapping of operational scenarios but also generated replanting path suggestions based on anomaly clustering analysis. 3. Improved YAN-YOLO11 Algorithm: A lightweight object detection algorithm was proposed, which significantly improved the recognition accuracy of transplanted seedlings in complex field environments while ensuring low computational consumption.

The remainder of this paper is organized as follows: Section 2 presents the architecture design of the online quality inspection method for tobacco transplanting operations. Section 3 focuses on the YAN-YOLO11 detection algorithm applied in transplanting quality inspection. Section 4 elaborates on the visualization and feedback functions of the digital twin system. Section 5 verifies the system’s performance and application effectiveness through experiments. Finally, Section 6 concludes the study and discusses directions for future optimization.

## 2 Digital twin system architecture

**Figure 1:**
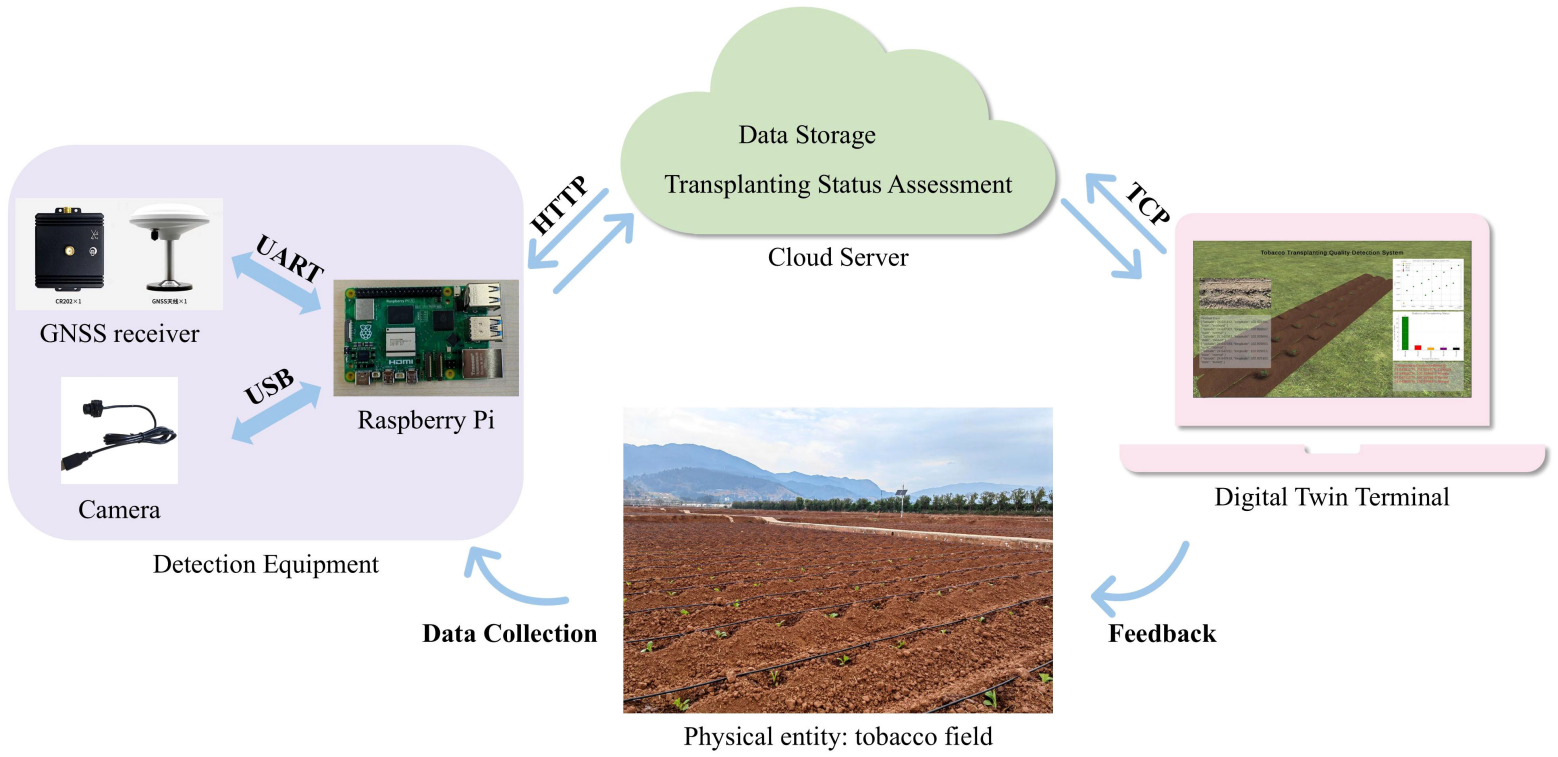
Overall framework of the digital twin system.

The architecture of the proposed deep learning- and digital twin driven tobacco transplanting quality inspection system is shown in **Figure 1**. The hardware components of the inspection system mainly include a CMOS camera, a GNSS receiver, and a Raspberry Pi 5. The system can be flexibly deployed on an independent mobile chassis or an automatic transplanter, provided that the sensors move along the center of the tobacco ridge to collect data. The hardware specifications in this study are as follows: The CMOS camera features a resolution of 1920×1080 pixels and a frame rate of 30fps. The GNSS receiver supports multi-frequency and multi-system operation (BDS/GPS/GLONASS/GALILEO), achieving a static horizontal positioning accuracy of ±1cm+1ppm and a vertical accuracy of ±2.5cm+1ppm.

As a distributed terminal, the Raspberry Pi connects to the camera via USB to capture video images and receives geographic location information from the GNSS receiver through a UART interface. Data preprocessing is carried out on the Raspberry Pi, including image enhancement of video frames and assigning timestamps to each frame, which are used to align data from different sensors through timestamp matching. The preprocessed data are then uploaded to the cloud server via the HTTP protocol.

The cloud server is responsible for transplanting status detection and assessment, data matching across different sensors, and data storage. A deep learning model is employed to detect tobacco seedlings in the video frames, and the transplanting status is then assessed based on the detection results. Data synchronization between the video frames captured by the CMOS camera and the geographic location information is achieved through strict system timestamp alignment.

On the digital twin side, the system adopts a client–server architecture based on the Transmission Control Protocol (TCP) (Mahmoodi Khaniabadi et al., 2023; Mishra and Sharma, 2023). Through the TCP, the system communicates with the cloud server to acquire transplanting status and geographic location information in real time. Using this information, it establishes virtual mappings of transplanted seedlings within the digital twin tobacco field, thereby achieving dynamic synchronization between the physical entities and the virtual scene.

The digital twin system not only displays transplanting quality status in the virtual tobacco field in real time but also generates alarms for abnormal transplanting cases and feeds the detected abnormal information back to the client. The feedback includes specific abnormal locations—such as missed planting points or root exposure points—as well as optimization suggestions. Through this feedback mechanism, the system forms a “collection–detection–mapping–feedback” closed loop, thereby improving the automation and precision of tobacco transplanting operations.

## 3 Deep learning driven transplanting quality inspection method

### 3.1 Dataset construction

**Figure 2:**
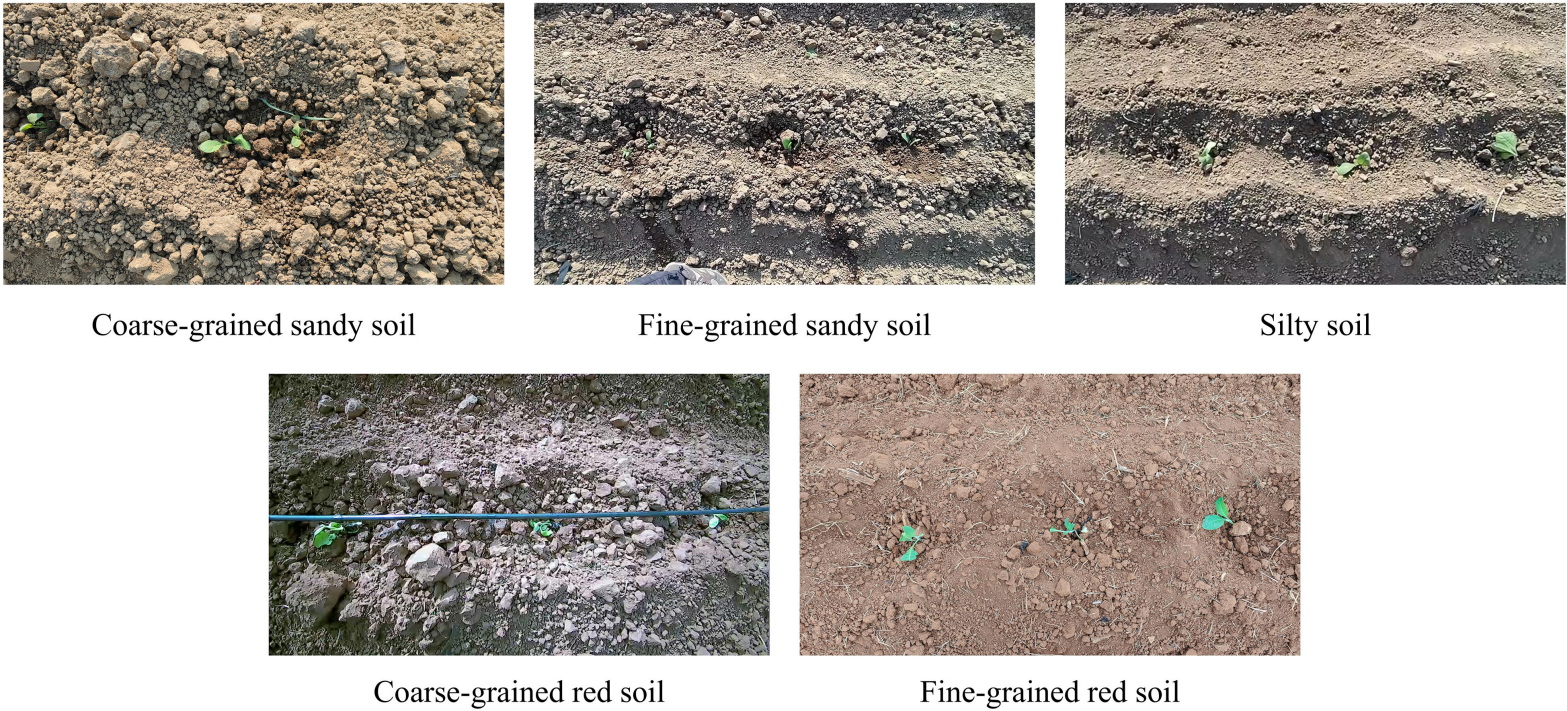
Typical examples of the dataset.

The dataset in this study was collected from tobacco fields in Mile City, Honghe Hani and Yi Autonomous Prefecture, and Chengjiang City, Yuxi, Yunnan Province. To ensure data diversity, the collection includes transplanting images under two different operation modes (manual and mechanical transplanting), lighting environments such as direct sunlight, backlight, and cloud shadows, and soil conditions (red soil and sandy soil with different particle sizes). Examples of images under different lighting and soil conditions are shown in **Figure 2**. The acquisition devices were a CMOS camera and a Huawei P50 smartphone. All videos were recorded at a resolution of 1920×1080 pixels, with a frame rate of 30 fps, and stored in mp4 and MOV formats. The collected videos were processed with a 1:50 frame extraction ratio, resulting in a total of 1,080 raw natural images.

To obtain clearer images with more distinct features and easier recognition, the raw natural images were preprocessed. Under natural lighting conditions, the illumination of tobacco fields is complex: transplanting pits and equipment occlusion can generate shadows, strong light can cause leaf reflections on transplanted seedlings, and on cloudy days the overall image contrast is relatively low. Therefore, contrast-limited adaptive histogram equalization (CLAHE) was employed to enhance the raw image data. The core idea of this method is to adaptively adjust the local contrast of images to improve visual quality, making it particularly suitable for images captured under complex lighting conditions (Reza, 2004; Kryjak et al., 2022).

**Figure 3:**
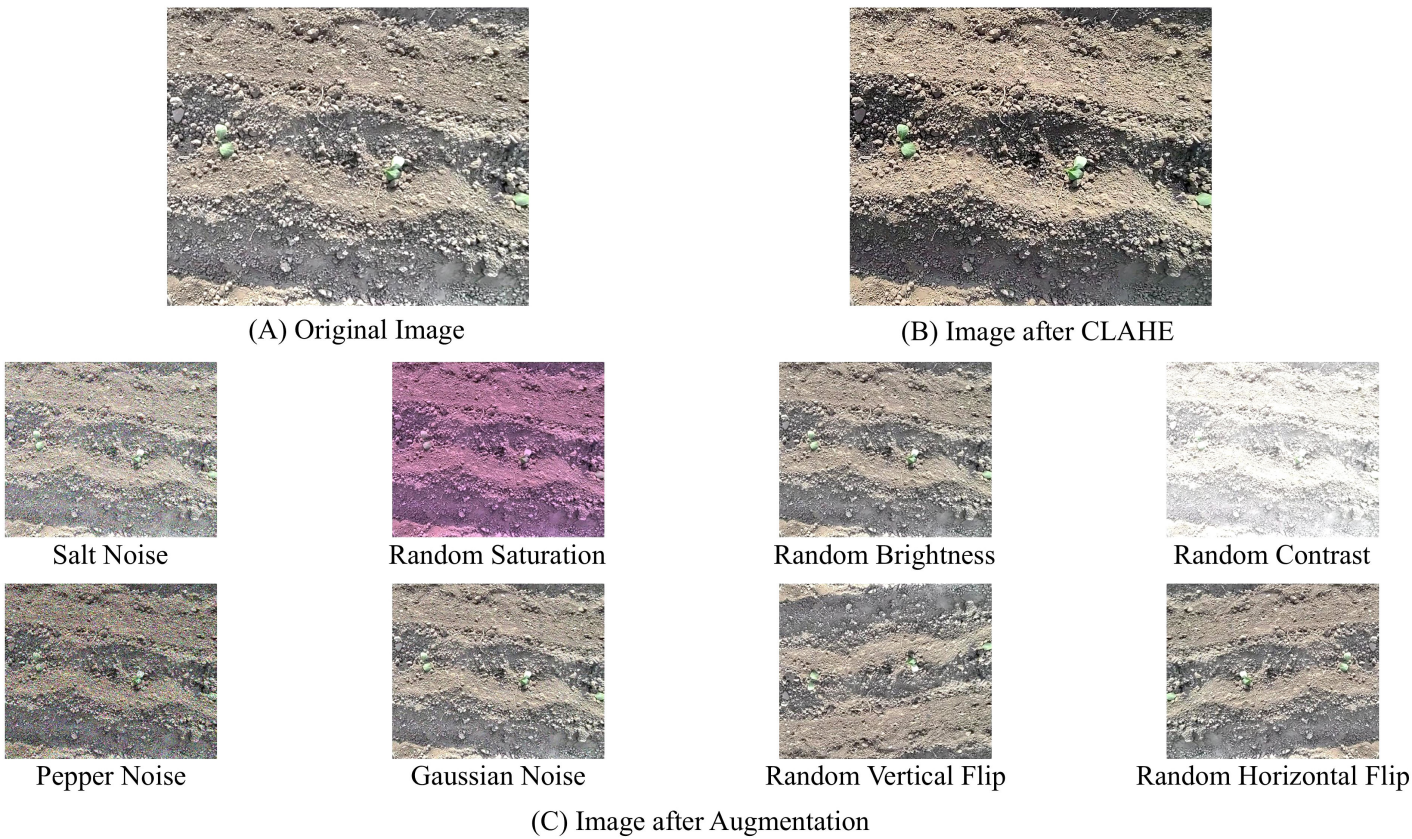
Dataset processing: **(A)** original image; **(B)** CLAHE preprocessing result; **(C)** data augmentation result.

A total of 1,080 images containing transplanted seedlings were divided into 865 for the training set and 215 for the validation set. The dataset was annotated using the LabelImg tool. To ensure the uniformity and accuracy of annotation standards, the dataset was annotated by a single researcher. Since the quantity of the three class labels in the collected raw image data was 8:1:1, with a small number of abnormal transplanted seedlings, data augmentation was applied to images containing abnormal seedlings to mitigate class imbalance and improve the model’s generalization and robustness (Wang et al., 2024). The augmentation methods included random flipping, random brightness, random contrast, random saturation, and random noise, as illustrated in **Figure 3**. After augmentation, the training set contained 2,764 images and the validation set 755 images, with 3,804 labels of normal transplanted seedlings and 3,047 labels of abnormal transplanted seedlings. The quantity of the three types of labels was adjusted to 2:1:1, resulting in a significant increase in the proportion of abnormal samples (Santos et al., 2022; Chen et al., 2024).

### 3.2 Improved YAN-YOLO11 detection algorithm

Owing to the requirements of the inspection system for real-time digital twin applications, real-time performance and accuracy are the key criteria for selecting and improving detection algorithms. In terms of model selection, RCNN and related models can achieve high-precision object detection; however, due to their use of multi-stage pipeline processing, they involve high computational cost and long processing time. Furthermore, compared with pixel-level segmentation models (e.g., DeepLab, U-Net), object detection frameworks such as the YOLO series adopt single-stage and end-to-end architectures. They can effectively capture transplanting status and coordinates without the overhead of dense prediction, possessing sufficient detection accuracy while meeting real-time requirements (Sapkota et al., 2024; Wang et al., 2025).

After comparing the performance of different YOLO versions on multiple public datasets, YOLO11 was found to achieve high accuracy with relatively low computational cost, making it an ideal choice for applications requiring both speed and precision (Jegham et al., 2024). The key improvements of YOLO11 include the introduction of the Cross-Stage Partial Self-Attention (C2PSA) module and the replacement of the C2f module with the C3k2 module, which enable more effective cross-layer contextual information capture and improve efficiency and speed with smaller kernels while maintaining accuracy (Sapkota et al., 2025).

**Figure 4:**
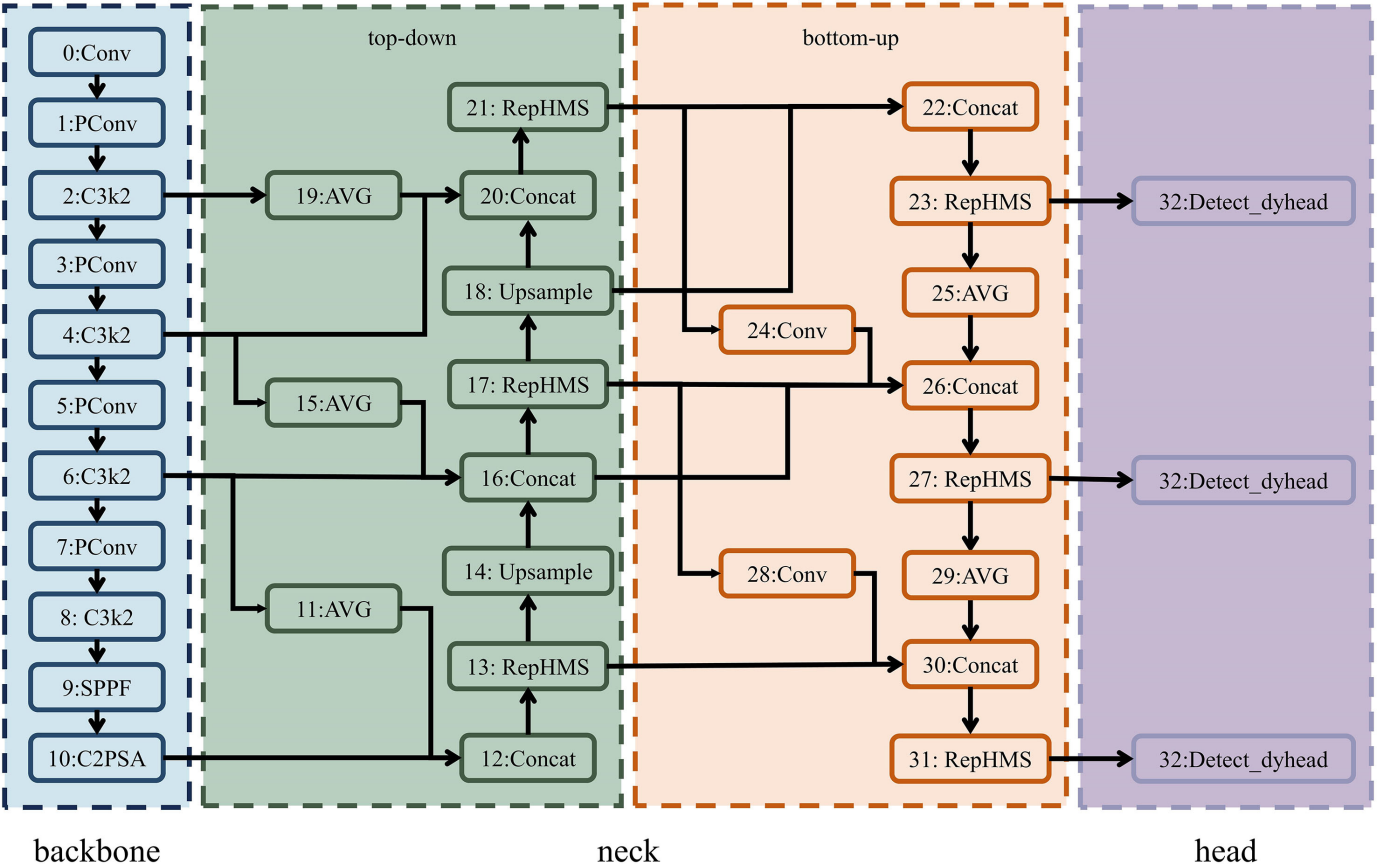
Network architecture of YAN-YOLO11.

Considering the requirements for accuracy and real-time performance in the transplanting detection system of this study, and taking into account the size characteristics of the target objects, we propose an improved YAN-YOLO11 detection algorithm, whose network architecture is illustrated in **Figure 4**.

In the detection head, to achieve lightweight design while maintaining high detection accuracy, Dynamic Convolution (Dynamic Conv) was introduced on top of the original decoupled head and depthwise separable convolution. Their combined effect reduces computational complexity and the number of parameters, while effectively preserving the model’s representational capacity. Traditional convolutional layers use a single kernel to process input features, whereas dynamic convolution introduces input-dependent coefficients *a* on top of the conventional convolution. A dynamic convolution with M kernels can be expressed as Equation 1:

(1) $$Y=\sum_{i=1}^{M}\alpha_{i}\;\left(W_{i}*X\right)$$

where *Y* is the output, *X* is the input, * denotes the convolution operation, $W_{i}$ is the *i*-th convolution weight, and $\alpha_{i}$ is the dynamic coefficient. The dynamic coefficients *a* are generated adaptively from the input *X*, by first applying global average pooling and then passing the result through a two-layer MLP module with softmax activation, i.e., Equation 2:

(2) α=softmax(MLP(Pool(X)))

Compared with conventional convolutional layers, the introduction of dynamic coefficients brings more parameters while incurring almost no additional computational overhead (Han et al., 2024). By incorporating dynamic coefficients, the dynamic convolution head can adaptively adjust convolutional weights, thereby enhancing the feature representation capability.

In the backbone network, to enhance the ability to analyze low-level features of small targets, the standard convolutions in the 1st, 3rd, 5th, and 7th layers were replaced with pinwheel-shaped convolutions (PConv). The pinwheel-shaped convolution module employs an asymmetric padding strategy to generate four convolution kernels in both horizontal and vertical directions, which process horizontal and vertical regions of the image separately. Through multiple convolutional operations, it processes the input feature map $X^{\left(h_{1},w_{1},c_{1}\right)}$, thereby enhancing the ability to capture target features. Its forward propagation process can be expressed as Equations 3–7:

(3) $$X_{1}^{\left(h^{\prime },w^{\prime },c^{\prime }\right)}=SiLU\left(BN\left(X_{P\left(1,0,0,3\right)}^{\left(h_{1},w_{1},c_{1}\right)}*W_{1}^{\left(1,3,c^{\prime }\right)}\right)\right)$$

(4) $$X_{2}^{\left(h^{\prime },w^{\prime },c^{\prime }\right)}=SiLU\left(BN\left(X_{P\left(0,3,0,1\right)}^{\left(h_{1},w_{1},c_{1}\right)}*W_{2}^{\left(3,1,c^{\prime }\right)}\right)\right)$$

(5) $$X_{3}^{\left(h^{\prime },w^{\prime },c^{\prime }\right)}=SiLU\left(BN\left(X_{P\left(0,1,3,0\right)}^{\left(h_{1},w_{1},c_{1}\right)}*W_{3}^{\left(1,3,c^{\prime }\right)}\right)\right)$$

(6) $${\text{X}}_{4}^{\left({\text{h}}^{\prime },{\text{w}}^{\prime },{\text{c}}^{\prime }\right)}=\text{SiLU}\left(\text{BN}\left({\text{X}}_{\text{P}(3,0,1,0)}^{\left({\text{h}}_{1},{\text{w}}_{1},{\text{c}}_{1}\right)}*{\text{W}}_{4}^{\left(3,1,{\text{c}}^{\prime }\right)}\right)\right)$$

(7) $$Y^{\left(h_{2},w_{2},c_{2}\right)}=SiLU\left(BN\left(Cat\left(X_{1}^{\left(h^{\prime },w^{\prime },c^{\prime }\right)},\ldots ,X_{4}^{\left(h^{\prime },w^{\prime },c^{\prime }\right)}\right)*W^{\left(2,2,c_{2}\right)}\right)\right)$$

Here, $W_{1}^{\left(1,3,c^{\prime }\right)}$ denotes a 1×3 convolution kernel with *c’* output channels; *P(1,0,0,3)* represents the number of padding pixels in the left, right, top, and bottom directions; Cat denotes the concatenation operation; and $W^{\left(2,2,C_{2}\right)}$ is the final normalization convolution kernel (Yang et al., 2025). Due to its unique padding scheme, pinwheel-shaped convolution forms a receptive field that attenuates outward in a manner similar to a Gaussian distribution. By adopting grouped convolution, it significantly enlarges the receptive field while minimizing the number of parameters (Luo et al., 2016; Zhang et al., 2017). When the kernel size is *k* = 3, the receptive field of a standard convolution is 9, whereas the pinwheel-shaped convolution expands it to 25 through the combination of four directional kernels, achieving a 177% increase.

In the neck structure, to address the issues of insufficient multi-scale feature fusion and inadequate small-object detection capability, a Multi-Branch Auxiliary Feature Pyramid Network (MAFPN) was introduced, which optimizes the feature processing pipeline through a bidirectional auxiliary fusion mechanism. In the structure, layers 11–21 form a top-down path that aggregates shallow high- and low-resolution layers with earlier features, enabling multidirectional gradient information exchange and enhancing the representation of medium- and large-scale objects. Layers 22–31 constitute a bottom-up path that fuses shallow backbone features with neck outputs, preserving shallow spatial information and providing richer details for small-object detection (Ke et al., 2024; Shi et al., 2025). To improve feature extraction efficiency, a Re-parameterized Heterogeneous Convolution (RepHMS) module was introduced into the neck feature processing. This module leverages a Global Heterogeneous Kernel Selection (GHSK) mechanism, which employs convolution kernels of different sizes across feature layers to capture multi-scale features ranging from local to global. In addition, through a re-parameterization mechanism, the multiple parallel multi-scale depthwise convolution kernels used during training are re-parameterized into a single kernel at the inference stage, thereby significantly enlarging the receptive field with almost no additional computational overhead (Yang et al., 2024).

### 3.3 Transplanting status assessment

The assessment and classification of transplanting status are at the core of this study. We propose a transplanting status assessment method based on seedling detection and positional information. By detecting normal, buried, and exposed-root seedlings in real time and combining GPS data with positional analysis, the method further assesses abnormal statuses such as double planting and missed planting. The transplanting statuses are prioritized in descending order as follows: Double Planting, Missed Planting, Seedling Burial, Root Exposure, and Normal Transplanting, and their assessment criteria are detailed in **Table 1**.

**Table 1:** Transplanting status assessment criteria.

Transplanting status	Spatial condition (d = spacing)	Visual detection (C = class)	Decision rule
Double Planting	d<0.4×d_std_	Ignored	Classified as Double Planting based on proximity, overriding any visual detection result.
Missed Planting	d>1.6×d_std_	Ignored	Classified as Missed Planting based solely on spatial gaps.
Seedling Burial	0.4×d_std_≤d ≤ 1.6×d_std_	Buried Seedling	If spacing is normal but the apical bud is covered with soil (detected as Buried Seedling), it is classified as Seedling Burial.
Root Exposure	0.4×d_std_≤d ≤ 1.6×d_std_	Exposed Root Seedling	If spacing is normal but the root substrate is exposed (detected as Exposed Root Seedling), it is classified as Root Exposure.
Normal Transplanting	0.4×d_std_≤d ≤ 1.6×d_std_	Normal Seedling	Classified as Normal Transplanting only when both spacing and visual status are normal.

Using the YAN-YOLO11 described earlier to process video frames, normal, exposed-root, and buried seedlings are detected. When a detection box passes through the center of the frame, the seedling is counted, and both the detected category and the timestamp of the frame are recorded. GNSS data are synchronized with video frames through timestamps, enabling the assignment of geographic coordinates (longitude and latitude) to each detected seedling. To calculate the in-row spacing of seedlings, the geographic coordinates must be converted into planar coordinates. Since the agronomic requirement for in-row spacing is generally around 0.5m, a simplified approximate planar projection method was adopted for rapid calculation within small areas. The conversion process is described in Equations 8–10:

*longitude direction:*

(8) x=longitude×meters_per_deg_lon

(9) meters_per_deg_lon =111000×cos(ref_lat)

*latitude direction:*

(10) y=latitude ×111000

Here, *meter_per_deg_lon* denotes the longitude coefficient, *ref_lat* is the reference latitude, and 111,000 is the approximate latitude conversion coefficient with units of *m/°*(meters per degree) (Snyder, 1997). The theoretical effective radius of the Local Tangent Plane (LTP) projection method (i.e., ENU coordinate conversion) can typically reach over 10km. The “small-scale area” defined in this study refers to fields with an operational area typically of 1km×1km (Drake, 2002). Using the converted planar coordinates, the Euclidean distance between adjacent seedlings is calculated and then compared with the agronomic standard spacing for further assessment. For missed planting, interpolation is used to calculate the coordinates of the missed-planting points. For double planting, consecutively detected seedlings with small in-row spacing are grouped as the same event, and the centroid coordinates are calculated to represent the double-planting point.

## 4 Digital twin design

In recent years, with the implementation of Agriculture 4.0, the exploration of digital twin technology in agricultural production practices has been deepening, gradually transforming areas such as agricultural production management and the optimization of agricultural machinery and equipment (Zhang et al., 2025). By employing high-fidelity models in virtual environments to achieve real-time mapping of physical entities, the field operation process can be perceived and identified in real time. This enables the evaluation of operational quality and feedback on detected issues, significantly enhancing the intelligence and refinement of agricultural production management (Verdouw et al., 2021). In this study, Unity3D was selected as the visualization platform for the digital twin system, with C# adopted as the primary programming language. The Unity platform offers powerful three-dimensional modeling and real-time rendering capabilities, supports efficient data interaction and cross-platform deployment, and provides flexible and scalable technical support for virtual simulation and visualization of tobacco transplanting (Lu et al., 2024).

### 4.1 Construction and mapping of virtual scenes

The digital twin platform establishes a stable connection via the TCP protocol with the transplanting status detection and assessment program deployed on the cloud server, enabling real-time data acquisition. The cloud server encapsulates the assessed results into JSON format for transmission. Each data entry corresponds to a transplanting point and contains transplanting status and geographic coordinates. An example is shown below:

*{“latitude”: 24.64190, “longitude”: 102.92841, “state”: “buried”}*.

Upon receiving the data, the digital twin converts the geographic latitude–longitude coordinates into a local East–North–Up (ENU) two-dimensional coordinate system for the virtual scene. The system employs a geographic coordinate projection conversion algorithm based on the origin of the tobacco field plot. The initial position of the detection system in the tobacco field is taken as the origin. Using the origin coordinates *(φ_0_, λ_0_)* and the clockwise angle *θ* between the ridge direction and true north as references, each transplanting point with geographic coordinates *(φ, λ)* is transformed into *(X, Z)* coordinates in the virtual scene. The conversion process is as follows (Equations 11–13):

(11) $$L_{\lambda }=R\cdot cos\varphi_{0}\cdot \left(\lambda -\lambda_{0}\right)\cdot \frac{\pi }{180}$$

(12) $$L_{\varphi }=R\cdot \left(\varphi -\varphi_{0}\right)\cdot \frac{\pi }{180}$$

(13) $$\left[\begin{array}{c} X\\ Z \end{array}\right]=\left[\begin{array}{cc} cos\theta & -sin\theta \\ sin\theta & cos\theta \end{array}\right]\left[\begin{array}{c} L_{\lambda }\\ L_{\varphi } \end{array}\right]+\left[\begin{array}{c} x_{off}\\ z_{off} \end{array}\right]$$

**Figure 5:**
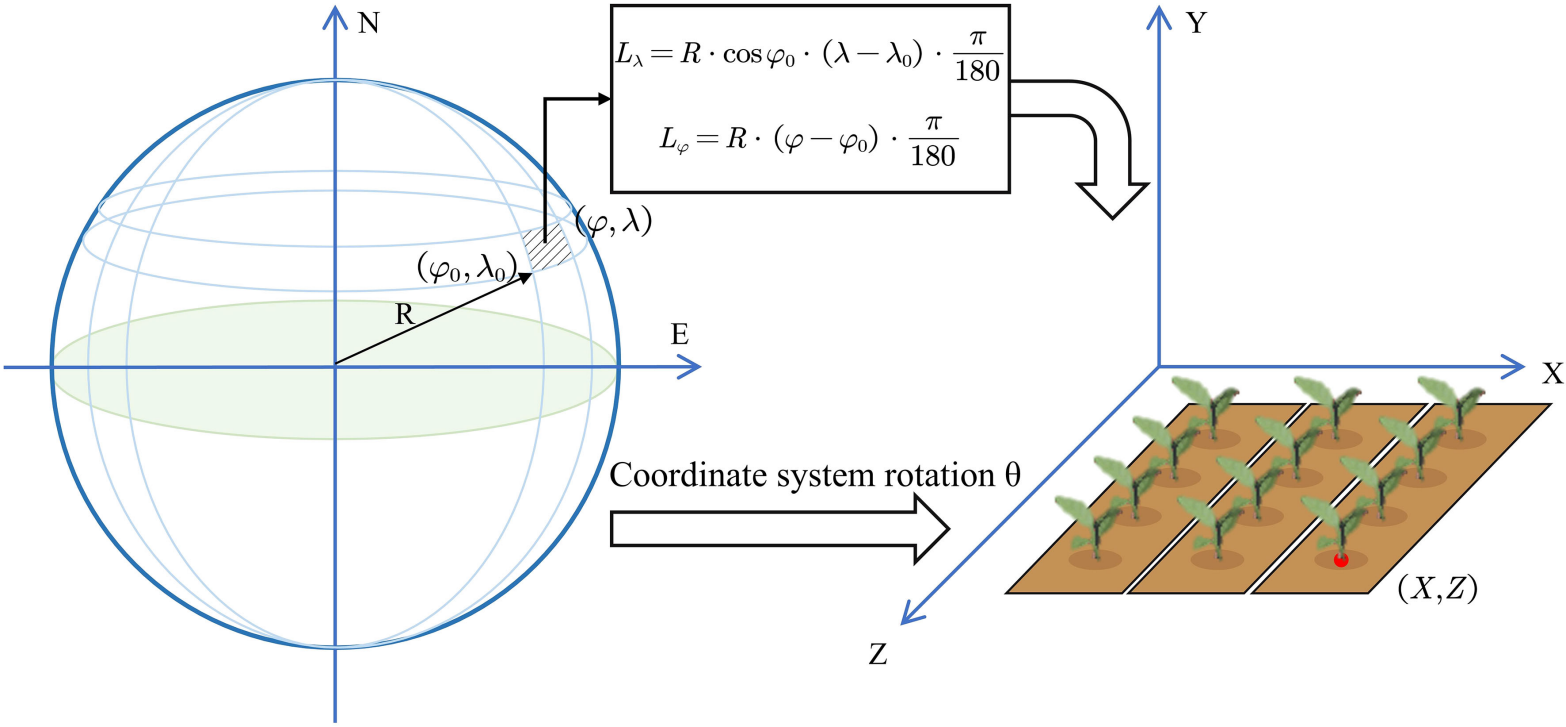
Coordinate transformation of transplanting points.

Here, *L_λ_* and *Lφ* denote the distances in the east–west and north–south directions. *R* is the semi-major axis radius of the WGS-84 ellipsoid, with *R*=6,378,137m. *x_off_* and *z_off_* represent the translation offsets along the horizontal and vertical axes, respectively (Teunissen and Montenbruck, 2017). Through this method, the transplanting points can be mapped into the virtual scene while preserving their actual relative spatial relationships, as illustrated in **Figure 5**.

**Figure 6:**
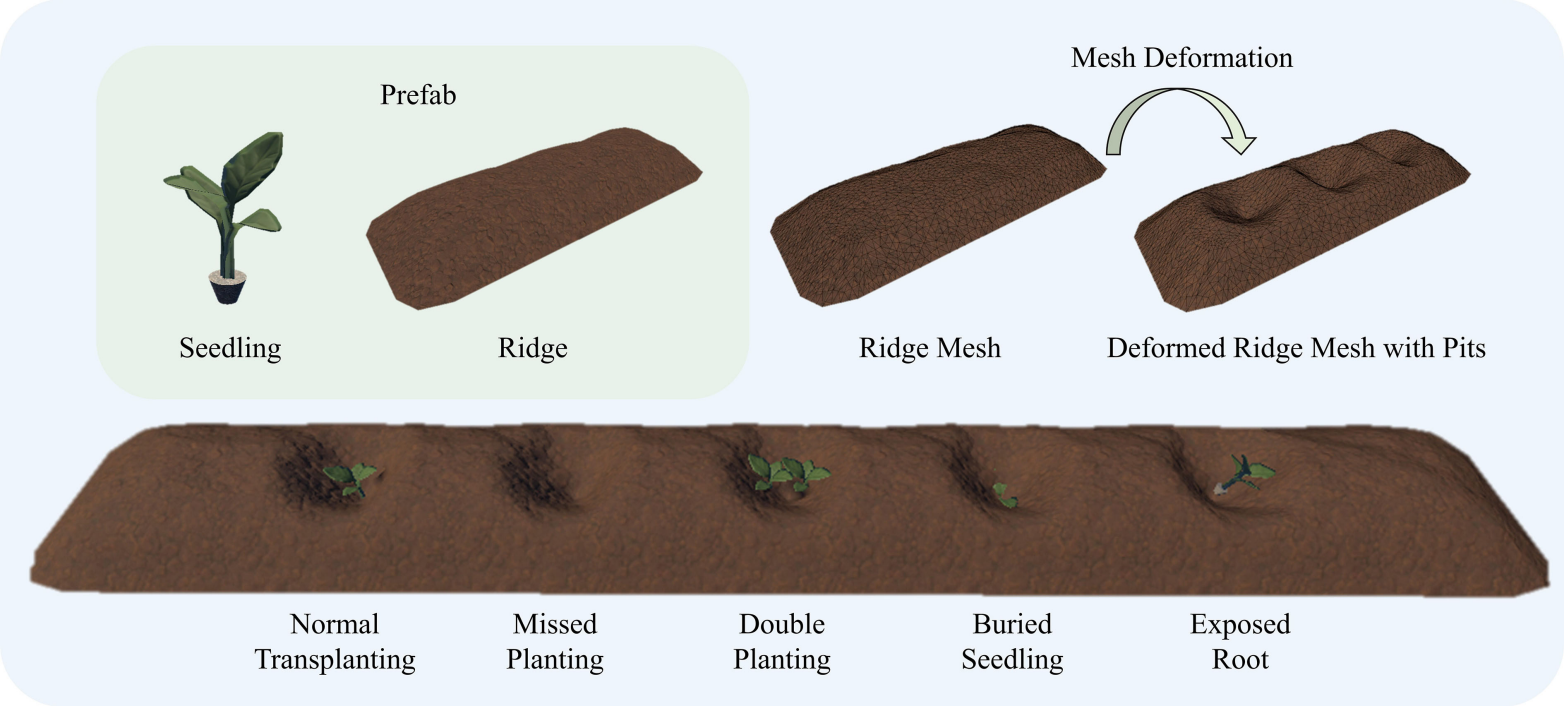
Modeling of five transplanting statuses.

In the transplanting status display of the virtual scene, ridge models are dynamically generated as prefabs through programmatic control. The three-dimensional structure of ridges is implemented using mesh grids, and transplanting pits are created through local mesh deformations to replicate the traces of transplanting operations. Based on the received transplanting status information, the system dynamically selects the number and placement of seedling models, thereby presenting the five transplanting statuses: normal, burial, root exposure, missed planting, and double planting. For example, in the normal transplanting status, one seedling model is placed at the center of each pit. In the root exposure status, the seedling model is shifted upward to expose part of the substrate. In the double planting status, two or more seedlings are placed within the same pit, with spatial distribution and angles adjusted to avoid overlapping. As shown in **Figure 6**, these operations enable the virtual scene to accurately reflect various transplanting statuses and spatial distribution characteristics, thus achieving fine-grained mapping and visualization from real tobacco fields to the virtual environment.

During the construction of the virtual scene, the system first generates ridge prefabs in batches according to field parameters and arranges them with specified width, length, and spacing, thereby accurately reproducing the structure of tobacco fields. As each transplanting data entry is received, the corresponding transplanting status in the virtual scene is updated in real time. To enhance performance under large-scale data, the platform adopts an object pooling mechanism and model simplification strategies, ensuring that the virtual scene runs smoothly even under complex operational conditions and meets the real-time requirements of intelligent field supervision.

### 4.2 Transplanting quality inspection and visualization

**Figure 7:**
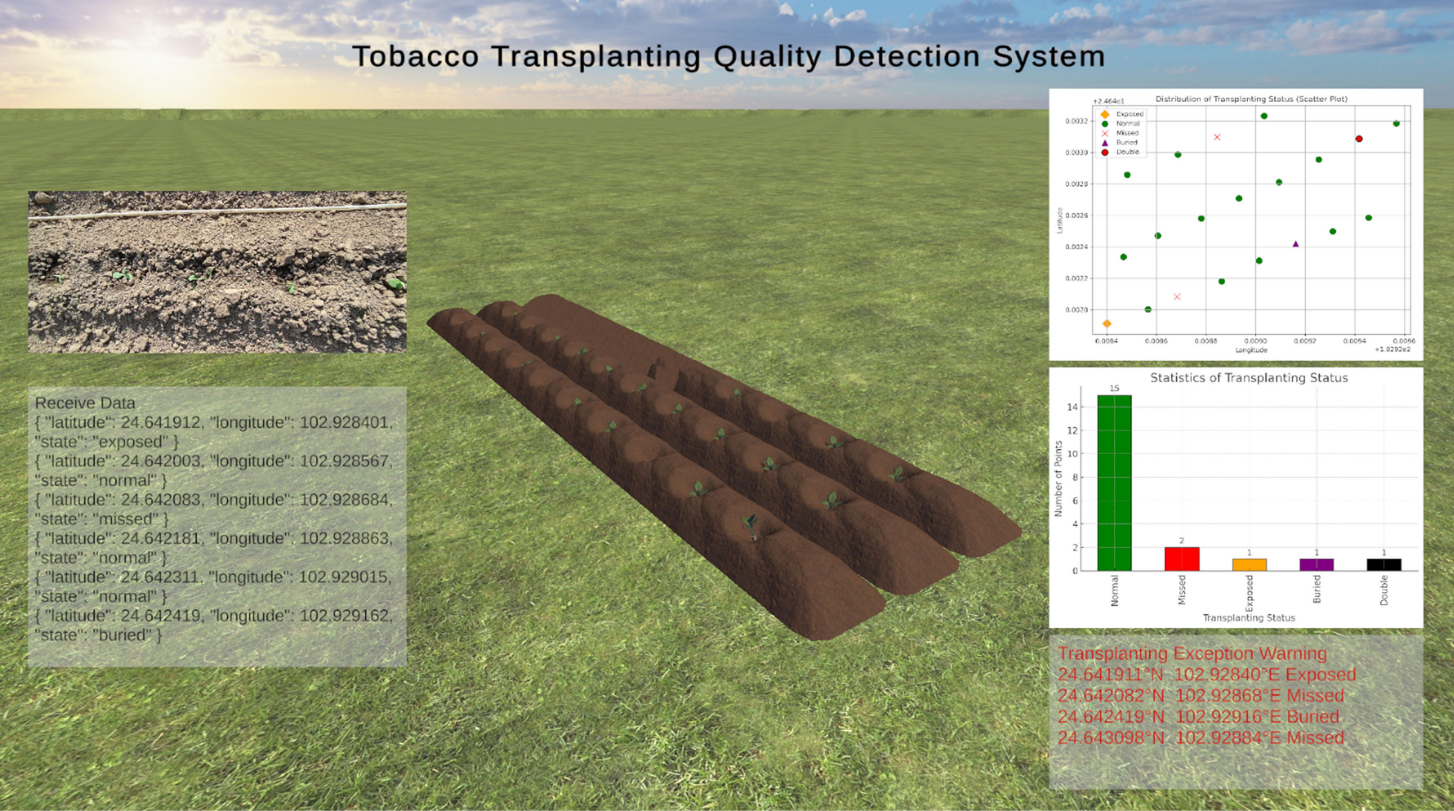
Digital twin interface of the tobacco transplanting quality inspection system.

This system leverages digital twin technology to provide visualization of transplanting quality inspection in virtual scenes, as well as abnormal transplanting alarms and replanting guidance. The system interface consists of multiple functional modules, including virtual entity display, physical entity images, data signal monitoring, transplanting data visualization, and replanting prompts, as shown in **Figure 7**.

In the virtual entity window, the system dynamically reproduces the actual operation of transplanting equipment through three-dimensional models based on the real-time received transplanting status and spatial location information, achieving high synchronization between physical operations and the virtual space. In the transplanting data visualization window, the platform continuously counts and updates the number and proportion of different transplanting statuses, including normal transplanting, missed planting, double planting, burial, and root exposure. For each abnormal transplanting status, the system quantifies it using dedicated indicators and automatically calculates the overall abnormality rate. The system employs bar charts to display the variations in quantity or proportion of each transplanting status, helping operators gain a comprehensive understanding of field operation quality. Replanting prompts are presented in the form of scatter plots, which intuitively mark all abnormal points on the spatial distribution map of the field, thereby facilitating the rapid localization and identification of problem areas.

### 4.3 Operational optimization feedback

In the design of the digital twin system, operational optimization feedback is not only a response mechanism for abnormal transplanting statuses but also a critical link for achieving dynamic interaction between the virtual scene and real-world tobacco field management. The system monitors transplanting statuses in real time, identifying four types of abnormal points—missed planting, double planting, root exposure, and burial—and generates replanting recommendation lists or optimal replanting paths based on their spatial clustering. This provides precise and timely decision support for field operation management.

The feedback mechanism functions through a dual-channel approach targeting both human operators and automated machinery. First, for manual intervention, the system transmits abnormal information in real time to the digital twin terminal. Through spatial clustering analysis of abnormal areas, it calculates the optimal replanting path and generates detailed suggestions, including the types of anomalies to be addressed. These outputs serve as intuitive guidance for manual replanting, enabling field managers to quickly locate problem areas.

Crucially, to achieve a fully interactive Digital Twin, the system extends this feedback to automated closed-loop regulation. By interacting with the control unit (ECU) of automatic transplanters, the system dynamically optimizes operational strategies. Specifically, utilizing the real-time recognition results, the system calculates deviation parameters and transmits control signals back to the machine. For adaptive depth adjustment, if a high frequency of “Buried Seedlings” or “Exposed Roots” is detected, the system sends a compensation signal to the depth control actuator (e.g., an electric push rod) to automatically raise or lower the planting mechanism. Similarly, for spacing calibration, feedback signals are generated to fine-tune the planting frequency or travel speed based on real-time spacing variations. This automated adjustment of key parameters not only improves replanting efficiency but also enhances equipment performance, achieving refined and intelligent transplanting operations.

Relying on this real-time synchronization, the operational optimization feedback mechanism enables a robust closed-loop interaction between the virtual and physical domains. The virtual scene functions not only as a visualization platform but as an active decision-making center. Through the closed-loop design of “collection-detection-mapping-feedback-execution,” the digital twin system ensures that dynamic adjustments are promptly transferred to real-world operations, effectively improving the automation and precision of tobacco transplanting.

## 5 Results and discussion

### 5.1 Performance comparison of different models

**Figure 8:**
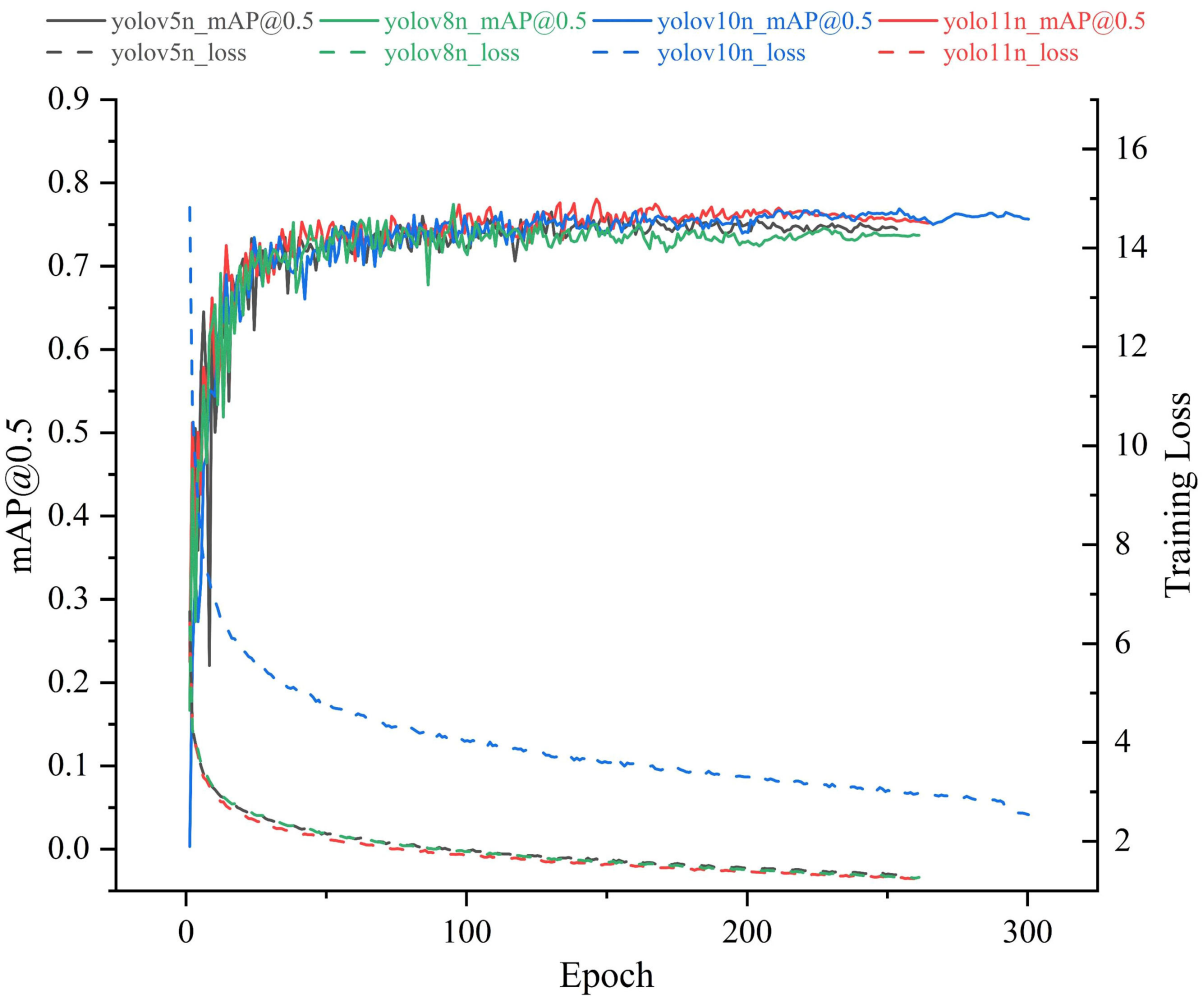
Training precision and loss curves of four models.

To achieve efficient detection of post-transplanting tobacco seedling images and to select the most suitable deep learning models for this task, lightweight versions of YOLOv5, YOLOv8, YOLOv10, and YOLO11 were trained and tested on the same post-transplanting image dataset under identical runtime environments and parameter settings, with the results shown in **Table 2** and **Figure 8**.

**Table 2:** Results of baseline model comparison test.

Model	P/%	R/%	mAP@50/%	mAP@50:95/%	Pa/M	FLOPs/G
YOLOv5n	81.0	72.8	75.3	43.6	1.9	4.5
YOLOv8n	84.2	73.1	77.0	43.6	3.2	8.7
YOLOv10n	82.3	76.2	77.5	46.8	2.3	6.5
YOLO11n	84.4	71.9	77.9	48.4	2.6	6.3

Based on the comparison of training results across the baseline models, it is evident that YOLO11n demonstrates the optimal balance between accuracy and efficiency. First, regarding the mAP@0.5 metric, YOLO11n performs comparably to YOLOv10n, with both outperforming YOLOv5n and YOLOv8n. Notably, its mAP@0.5:0.95 surpasses that of all other models. This superiority is attributed to YOLO11’s enhanced feature extraction capabilities; its improved backbone and neck architectures enable more precise capture of tobacco seedling features. Second, in terms of Loss curves, YOLOv10n exhibits significantly higher loss values than the other models, indicating greater difficulty in optimization within complex scenarios and a failure to effectively fit the data. In contrast, YOLO11n demonstrates the lowest loss values and the fastest convergence speed. This validates YOLO11’s optimized training pipeline and architectural design, allowing it to rapidly minimize prediction errors and maintain exceptional training stability, even with fewer parameters.

In summary, YOLO11n not only inherits the generational advantage of “fewer parameters and higher precision” but also excels in training convergence and robustness. Its strong adaptability to edge computing environments, combined with its capability for feature extraction in complex backgrounds, fully satisfies the stringent requirements of this tobacco transplanting quality inspection system for high precision, real-time performance, and stability. Therefore, selecting YOLO11n as the baseline model for improvement in this project is the optimal choice.

### 5.2 Evaluation and ablation study of YAN-YOLO11

**Figure 9:**
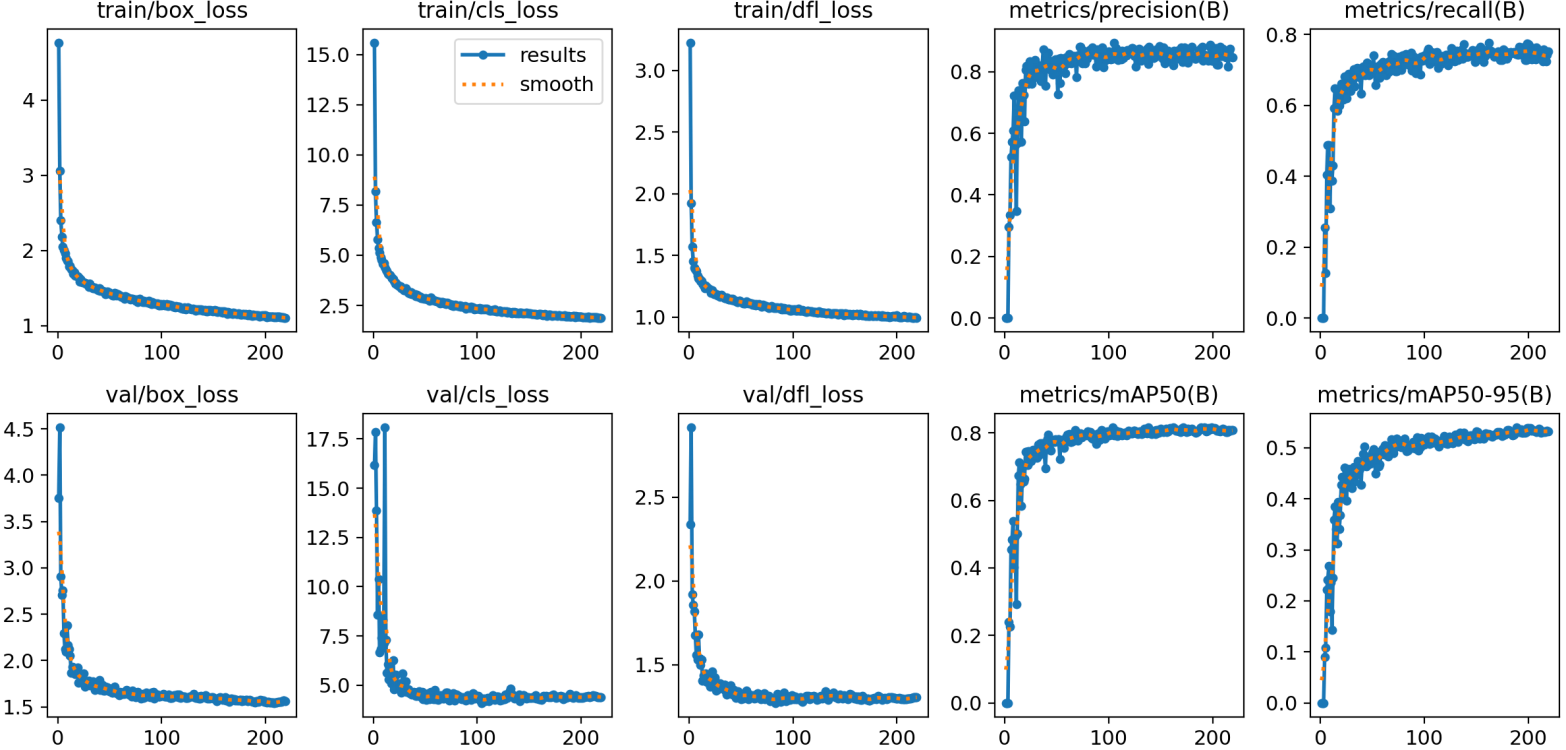
Training and validation performance curves of the YAN-YOLO11 model.

The model training configuration for this study is as follows: The AutoDL cloud computing platform was selected, equipped with an NVIDIA GeForce RTX 4090 GPU, a 20 vCPU Intel Xeon Platinum 8470Q processor, and 64 GB of RAM. The operating system was Linux, and the deep learning framework employed was PyTorch 2.4.1, running on Python 3.8.10 and CUDA 11.8. Regarding hyperparameters, the initial learning rate was set to 0.01, the batch size was set to 16, and the AdamW optimizer was selected. The model was trained for 300 epochs with an early stopping patience of 30 epochs, and the input image size was set to 640 × 640 pixels. The training results are shown in **Figure 9**.

To further verify the effectiveness of the improvements in YAN-YOLO11, ablation experiments were conducted, and the results are presented in **Table 3**. Analysis of the results shows that the improved dynamic convolution detection head plays a significant role in model lightweighting, while the enhancements in pinwheel-shaped convolution and the neck structure substantially improve model accuracy (Bochkovskiy et al., 2020). Compared with the original YOLO11, the improved YAN-YOLO11 achieved increases of 2.4% in Precision and 2.5% in Recall. The mean average precision (mAP) at an IoU threshold of 0.5 and at IoU thresholds of 0.5-0.95 improved by 3% and 5.8%, respectively, while the number of parameters and floating-point operations (FLOPs) were reduced by 19.8% and 9.5%.

**Table 3:** Results of ablation experiments.

Dyhead	PConv	MAFPN	P/%	R/%	mAP@50/%	mAP@50:95/%	Pa/M	FLOPs/G
–	–	–	84.4	71.9	77.9	48.4	2.58	6.3
✓	–	–	81.8	76.5	77.5	48	2.38	4.9
–	✓	–	86.1	74.7	80.5	52.9	2.54	6.9
–	–	✓	83.8	72.5	79.3	50.1	2.10	6.1
✓	✓	–	84.1	72.4	79.6	51.3	2.33	5.4
✓	–	✓	85.6	74.4	80.5	52	2.13	5.6
–	✓	✓	84.3	74	80.1	52.2	2.24	7.1
✓	✓	✓	86.8	74.4	80.9	54.2	2.07	5.7

To evaluate the robustness of the model, we conducted multiple experiments on YAN-YOLO11 using different random seeds and calculated the mean and Standard Deviation (SD) for key metrics. The standard deviations for the key metrics of YAN-YOLO11 are as follows: Precision is 86.8% ± 1.7%, Recall is 74.4% ± 2.7%, mAP@50 is 80.9% ± 1.4%, and mAP@50:95 is 54.2% ± 1.0%. These values reflect the stability and magnitude of improvement of the model across multiple runs. We added 95% Confidence Intervals (CI): The CI for Precision is [86.0%, 87.6%], for Recall is [73.4%, 75.4%], for mAP@0.5 is [80.0%, 81.8%], and for mAP@0.5:0.95 is [53.0%, 55.4%]. Furthermore, we verified the significance of these improvements using a Binomial test (assuming the baseline corresponds to the values of the original YOLO11n). All p-values were less than 0.05, indicating that the improvements are statistically significant.

**Figure 10:**
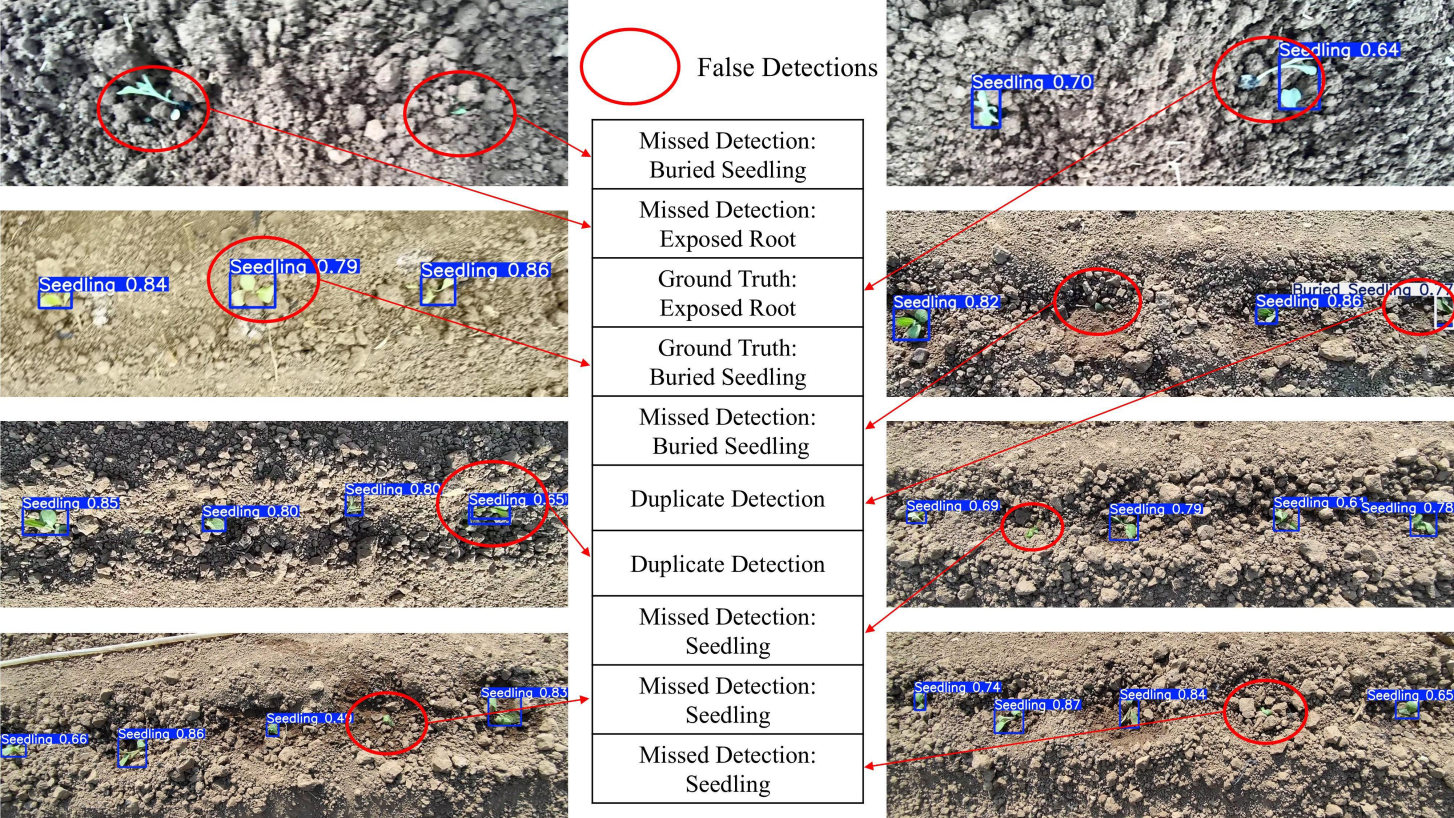
Analysis of typical detection failure cases in field scenarios.

To further analyze the robustness and limitations of the system in complex scenarios, **Figure 10** illustrates typical recognition results, particularly typical recognition errors. In the examples of correct detections, the system demonstrates strong robustness under complex lighting conditions such as strong backlighting and cloud shadows. This is attributed to the CLAHE preprocessing and the enhanced feature extraction capabilities of the YAN-YOLO11 backbone, which effectively suppress environmental interference. However, false identifications, missed detections, or duplicate detections still occur in certain extreme scenarios. “Misclassification” errors (e.g., classifying exposed-root seedlings as normal seedlings) typically occur when the root area is visually ambiguous due to soil clumps. “Missed detections” mainly occur when seedlings are severely occluded by soil (deeply buried) or when the leaf area is extremely small. In future work, we plan to integrate 3D depth information and temporal information. By employing sequence analysis, we aim to resolve these visual ambiguities and further reduce false detections.

Overall, the improvements enhanced detection accuracy while further optimizing model lightweighting, demonstrating that the proposed algorithm performs effectively in detecting post-transplanting tobacco seedlings.

### 5.3 System verification experiments

**Figure 11:**
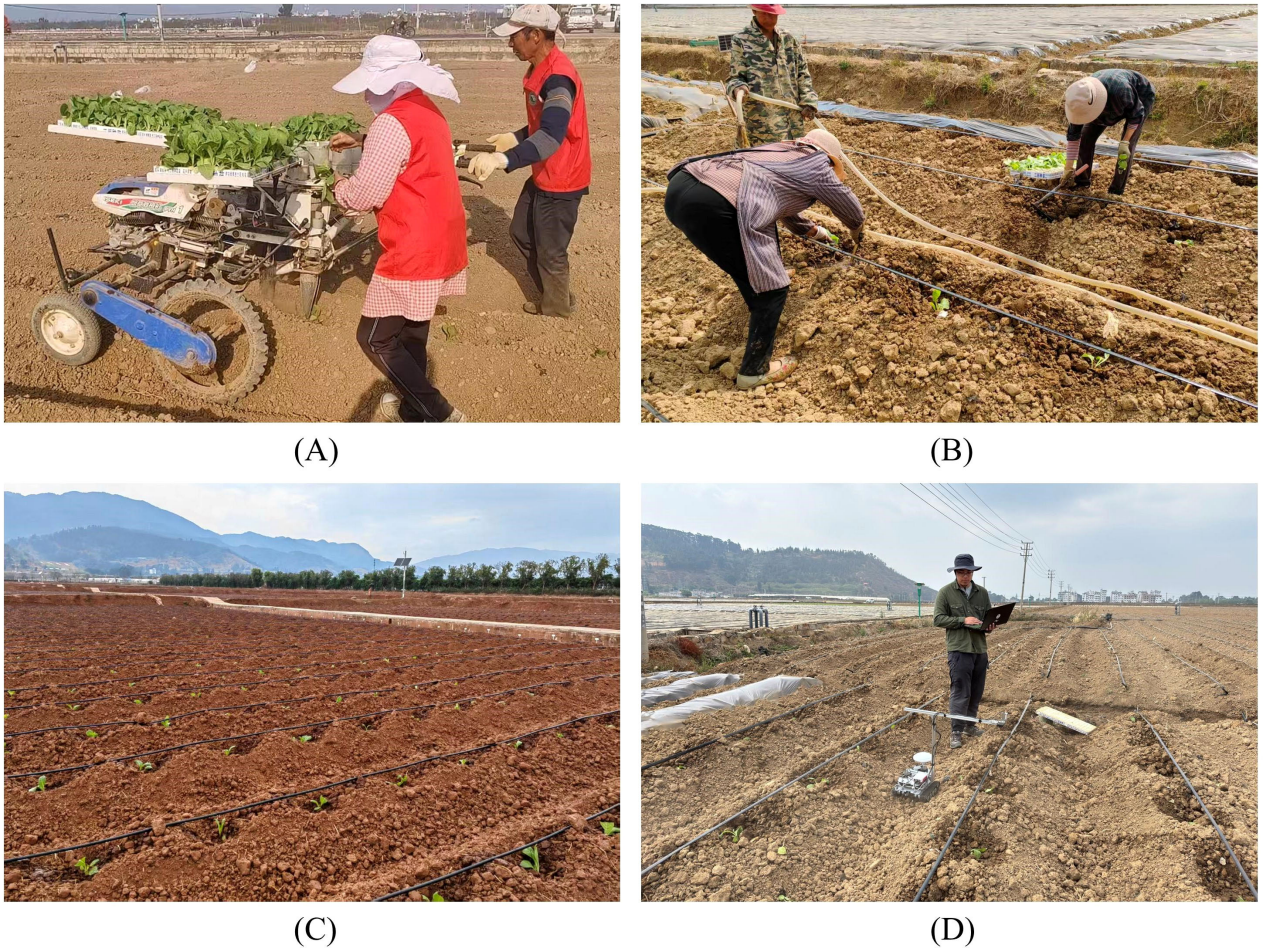
**(A)** Semi-automatic transplanting machine operation site; **(B)** manual transplanting operation site; **(C)** tobacco field after transplanting; **(D)** experimental validation environment.

To verify the functionality and performance of the online detection system, field experiments were conducted at the tobacco fields in Chengjiang City, Yuxi, Yunnan Province and Mile City, Honghe Hani and Yi Autonomous Prefecture, with the hardware system mounted on a self-propelled crawler chassis, as shown in **Figure 11**. The Chengjiang experimental plot consisted of 10 ridges, each with 21 theoretically transplanted seedlings, totaling 210 theoretical transplanting points; the Mile experimental plot consisted of 8 ridges, each with 48 theoretically transplanted seedlings, totaling 384 theoretical transplanting points. The two plots comprised a total of 594 theoretical transplanting points. The experimental results show that the system accurately identified transplanting points and their statuses, achieving 539 correct determinations and a transplanting status accuracy of 90.74%, as detailed in **Table 4**. The related demonstration video and code has been made publicly available on GitHub: https://doi.org/10.5281/zenodo.17075402.

**Table 4:** Results of system field validation experiments.

Transplanting status	Ground truth	Assessment results	Correct	Accuracy(%)
Normal Transplanting	503	495	463	92.05
Root Exposure	16	13	13	81.25
Seedling Burial	13	14	10	76.92
Missed Planting	51	57	45	88.24
Double Planting	11	10	8	72.73
Total	594	589	539	90.74

To evaluate the statistical stability of the system’s accuracy, we conducted a statistical analysis on the overall transplanting status assessment accuracy. The 95% Confidence Interval (CI) was calculated using the Wilson Score Interval method, determining that the system’s true accuracy falls within the range of [88.14%, 92.82%]. This interval confirms that, under the current sample size, the reported results possess high statistical reliability. Furthermore, to validate the effectiveness of the system’s performance, we conducted significance tests using 85% as the baseline. The p-value for the Binomial test was 2.16e-05, and the p-value for the Z-test was 4.46e-05; both are significantly less than 0.05. This demonstrates that our system is effective and holds practical application value.

During the experiment, the digital twin system realized end-to-end integration of model detection, spatial mapping, and virtual scene visualization, enabling the acquired transplanting images and data to be transmitted and intuitively reflected in the virtual scene in real time. During operation, the system continuously received detection results and transplanting status judgments from the YAN-YOLO11 model, covering five statuses: normal transplanting, missed planting, double planting, buried seedlings, and exposed-root seedlings. Through latitude–longitude coordinate transformation, all detected points were accurately mapped to the corresponding positions in the virtual tobacco field, achieving dynamic synchronization between physical entities and virtual entities. The system also integrated bar charts, scatter plots, and other statistical and visualization tools to automatically display the numbers and distributions of various transplanting statuses.

In terms of system performance, we adopted a distributed architecture: the Raspberry Pi serves solely as an edge device responsible for data acquisition, preprocessing, and transmission, while the core YAN-YOLO11 model inference is deployed on a cloud server. The YAN-YOLO11 model achieved an average inference speed of 30.66 FPS on the cloud server equipped with an NVIDIA RTX 4090 GPU, sufficient for stable and smooth real-time analysis of video frames and status output. To verify the real-time performance and stability principles of the digital twin system design, the Unity engine performance analysis tool Profiler was used to analyze scene performance during system operation. With continuous reception and rendering of multiple types of point data, the platform achieved an average per-frame time of 33ms and an average frame rate of 30FPS. During testing, the digital twin platform demonstrated excellent load-bearing capacity and smooth interactive response, with all transplanting point positions and statuses accurately reproduced in the scene.

To quantify the real-time synchronization performance of the digital twin system, we analyzed the end-to-end latency, which primarily consists of three components: data transmission latency, model inference and data fusion latency, and virtual scene rendering and feedback latency. Specifically, the average latency for capturing images and GNSS data from the edge device (Raspberry Pi) and transmitting them to the cloud server for model inference is 20ms. The average time required to complete YAN-YOLO11 inference, GNSS data fusion, and transplanting status assessment on the cloud server (RTX 4090) is 32.6ms. Furthermore, in the Unity digital twin platform, the average latency for receiving all computational results and rendering the updated virtual scene is approximately 33ms. Consequently, the total end-to-end synchronization latency is approximately 85.6ms. This latency is significantly lower than the time interval between two tobacco seedlings at a typical transplanter speed of 0.5m/s, ensuring that the digital twin system is capable of achieving quasi-real-time monitoring and feedback.

**Figure 12:**
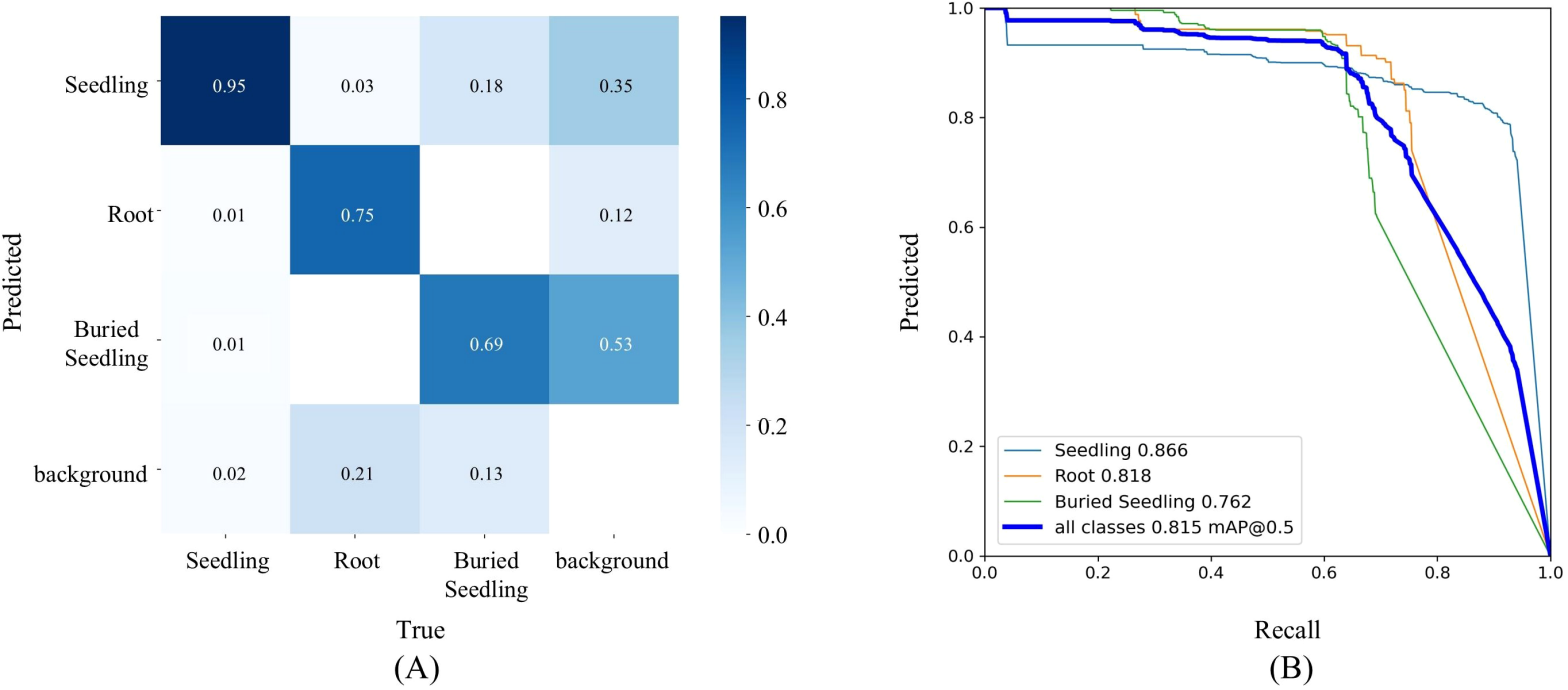
**(A)** Confusion matrix of YAN-YOLO11; **(B)** precision–recall curves.

Regarding the recognition results of YAN-YOLO11 in the validation, for the three target categories—normal transplanting, buried seedlings, and exposed-root seedlings—**Figure 12** shows that the recognition accuracy of the model for normal transplanting seedlings was significantly higher than for exposed-root and buried seedlings. The correct prediction rate for normal seedlings reached 95.20%, with both precision and recall being high, and the curve approaching the upper-right corner, indicating excellent recognition performance for this category. In contrast, the recognition accuracy for the other categories was relatively lower, with noticeable misclassification between exposed-root and buried seedlings. In actual transplanting operations, the occurrence probabilities of buried and exposed-root seedlings are much lower than those of normal transplanting, double planting, and missed planting. As a result, the overall recognition accuracy verified in the experiment was significantly higher than the performance of YAN-YOLO11 during training, reaching an accuracy of 90.74%. This result indicates that the model’s high accuracy for normal transplanting seedlings helped improve the overall accuracy, while misclassification of exposed-root and buried seedlings was partially alleviated.

The impact of recognition errors on status assessment was also validated during the experiment. Recognition results directly influence transplanting status judgment. When a missed detection occurs, the distance between adjacent seedlings increases due to the missed detection, causing the missed point to be classified as missed planting. In the case of a false detection, the ground or other background may be mistakenly identified as a transplanting seedling, resulting in a decrease in the distance between it and other correctly identified seedlings, leading to a false classification of double planting. Misclassification of categories does not affect the proportions of missed or double planting. Therefore, the overall transplanting point and status judgment accuracy should be similar to the recognition accuracy.

Overall, the improved YAN-YOLO11 network demonstrated high accuracy in recognizing normal, exposed-root, and buried transplanting seedlings. Combined with geographic location information, it reliably and stably detected double planting and missed planting events, yielding results highly consistent with manual inspection. The system exhibited excellent real-time performance, low status update latency, and met the requirements for field operations.

### 5.4 Limitations and future work

Although the deep learning and digital twin-based online inspection system proposed in this study meets practical application requirements in terms of accuracy and real-time performance, we objectively acknowledge several limitations in the current work, which also highlight directions for future research.

First, the dataset exhibits limited variations, as it was primarily collected from two locations in Yunnan Province under two main soil types (red soil and sandy soil) and specific weather conditions (sunny and cloudy). While data augmentation techniques were employed to address class imbalance for abnormal states (e.g., buried and exposed-root seedlings), the model’s robustness to these synthetically generated features remains inferior to that achieved with real samples. This constraint may affect the system’s performance in diverse geographical regions, soil compositions, tobacco varieties, or more extreme environmental conditions, such as heavy rain or dense fog, where lighting and occlusion variations could be more pronounced. The transferability of the model across these broader scenarios has not been fully validated. Future efforts will involve expanding the dataset through extensive field collections to include larger-scale, more diverse real abnormal samples, as well as incorporating cross-regional and cross-variety migration tests. Additionally, advanced techniques like domain adaptation, zero/few-shot learning, or the generation of synthetic data (e.g., using generative adversarial networks to simulate varied lighting, occlusion, and environmental scenarios) could enhance the model’s generalization and environmental adaptability.

Second, the system has notable computational requirements, particularly for edge deployment on resource-constrained devices like the Raspberry Pi. Although real-time processing (approximately 30 FPS) is achieved on the cloud server, edge-based inference demands high computational power, which could limit scalability in low-power field environments. This is compounded by potential synchronization delays between virtual and physical models in the digital twin framework, for which quantitative metrics (e.g., latency measurements) are currently lacking. Future optimizations could explore lighter model architectures, model compression techniques (e.g., pruning or quantization), or dedicated edge hardware such as the NVIDIA Jetson series to improve energy efficiency and enable higher-frequency real-time closed-loop control.

Third, the system’s effectiveness depends heavily on precise sensor calibration, including the alignment of the CMOS camera, GNSS receiver, and timestamp matching across data streams. Any misalignment or drift in sensor calibration—due to vibrations during field operations, environmental interference, or hardware inaccuracies—could introduce errors in multi-sensor data fusion, in-row spacing estimation, and transplanting status assessment. This reliance underscores the need for robust calibration protocols in practical deployments. To mitigate this, future work could integrate automated calibration mechanisms or fault-tolerant sensor fusion algorithms.

In addition to addressing these limitations, potential improvements include transitioning to transformer-based detectors (e.g., DETR or Vision Transformers), which could better handle complex occlusion and lighting conditions by leveraging attention mechanisms for global context understanding, potentially surpassing the convolutional focus of YOLO models. Furthermore, enhancing the digital twin with 3D simulations—incorporating depth data from additional sensors could provide more immersive visualizations and predictive capabilities for transplanting scenarios, enabling advanced what-if analyses for operational optimization.

## 6 Conclusion

This study addresses the quality inspection of tobacco transplanting and the optimization of field operations. We proposed and implemented an online quality inspection method that integrates deep learning driven detection with digital twin visualization. Leveraging the improved YAN-YOLO11 model and multi-sensor data fusion, the system achieves accurate transplanting status detection and assessment, as well as spatial localization and dynamic visualization of abnormal cases. In summary, the main conclusions are as follows:

1. Transplanting Status Detection: The proposed lightweight improved YAN-YOLO11 algorithm can achieve high-precision detection of normal seedlings, buried seedlings, and exposed-root seedlings. Compared with YOLO11n, its precision and recall increased by 2.4% and 2.5%. The mean average precision (mAP@0.5 and mAP@0.5:0.95) increased by 3% and 5.8%. At the same time, the model complexity was significantly reduced, achieving a balance between accuracy and real-time performance.
2. Multi-sensor Data Fusion: By fusing GNSS positioning data with visual detection results, the system achieves high-precision estimation of in-row spacing and assessment of missed planting and double planting. In field experiments, the accuracy of transplanting status assessment reached 93.81%, which was highly consistent with manual inspection results, demonstrating the stability and reliability of this module.
3. Digital Twin Visualization: A virtual tobacco field was constructed on the Unity3D platform to achieve three-dimensional dynamic mapping and real-time visualization of detection results. The system maintained a stable frame rate of about 30 FPS, which allowed it to clearly reflect the spatial distribution of transplanting points and enhance the intelligence of field operations.
4. Operational Optimization Feedback: The system can generate pending replanting lists and optimal replanting routes based on the spatial clustering of abnormal points, providing intuitive guidance for operators. At the same time, the feedback serves as data support for the optimization of transplanting equipment operational parameters. It establishes a “collection-detection-mapping-feedback” closed loop and effectively improves both operational efficiency and equipment performance.

Overall, the digital twin system for tobacco transplanting quality inspection developed in this study markedly enhanced the digitalization and refinement of transplanting operations, aligned with the vision of Agriculture 4.0, and accelerated the intelligent transformation of tobacco production management. With the continuous improvement of algorithms and platforms, this system will be further refined for applications in complex environments, such as large-scale multi-plot fields and multi-device access. It will be extended to a broader range of crop types and field operational stages, facilitating the deep integration of agricultural production with digital twin systems and enhancing its practical application in commercial projects.

## Data Availability

The datasets presented in this study can be found in online repositories. The names of the repository/repositories and accession number(s) can be found in the article/supplementary material.
