# Correction: A digital twin–driven deep learning framework for online quality inspection in tobacco transplanting

**DOI:** 10.3389/fpls.2026.1804394

**Published:** 2026-02-17

**Authors:** Qiuyang Zhao, Erdeng Ma, Jian Zhao, Zekun You, Jiahui Liu, Dong Zhao

**Affiliations:** 1 School of Technology, Beijing Forestry University, Beijing, China; 2 Yunnan Academy of Tobacco Agricultural Sciences, Kunming, China; 3 Key Lab of State Forestry Administration on Forestry Equipment and Automation, Beijing Forestry University, Beijing, China; 4 State Key Laboratory of Efficient Production of Forest Resources, Beijing, China

**Keywords:** crop management, deep learning, digital-twin, online quality inspection, tobacco, transplanting

In the published article, there was a mistake in the **Funding** statement. The section originally stated “The author(s) declared that financial support was received for this work and/or its publication. The authors declare that this study received funding from China National Tobacco Corporation Yunnan Province Branch. The funder was not involved in the study design, collection, analysis, interpretation of data, the writing of this article or the decision to submit it for publication.”

The corrected statement is: “The author(s) declared that financial support was received for this work and/or its publication. The authors declare that this study received funding from China National Tobacco Corporation Yunnan Province Branch, under the major project of the Scientific and Technological Plan of Yunnan Tobacco (project number: 2024530000241018). The funder was not involved in the study design, collection, analysis, interpretation of data, the writing of this article or the decision to submit it for publication.”

The original version of this article has been updated.

